# Party branches, policy perception and corporate social responsibility: Evidence from Chinese private enterprises

**DOI:** 10.3389/fpsyg.2022.1048060

**Published:** 2023-01-06

**Authors:** Zhenjiu Yao, Zengtian Zhang, Jun Ma

**Affiliations:** ^1^School of Management, University of Science & Technology of China, Hefei, China; ^2^School of Business, Jiangnan University, Wuxi, China

**Keywords:** party branches, private enterprises, corporate social responsibility, policy perception, China

## Abstract

**Introduction:**

Party branches embedded in private enterprises are a unique phenomenon in the Chinese economy, but few studies have focused on the economic consequences. We hope to explore the impact of party branches on small and medium-sized private enterprises’ corporate social responsibility (CSR) in China in order to fill the gaps in current research.

**Methods:**

Data were used from the 11th Chinese Private Enterprise Survey (CPES) in 2014. The study uses the methodology of fixed effect model, mediation analysis and moderation analysis. Moreover, propensity score matching and Heckman two-step method deal with the endogeneity problem and check the robustness of the results.

**Results:**

We find that, first, the embedding of party branches will improve the CSR performance of private enterprises in various dimensions by enhancing the perception of private enterprises in policy; second, in various influence pathways, the party branches will enhance the perceptions of policy related to economic interests, which has a more significant impact on enhancing the performance of philanthropic CSR. Further research reveals that business owners’ first-given and later-generated political connections support the party branches’ perception of policies related to economic and social interests, respectively.

**Discussion:**

The findings suggest that political intervention by China’s ruling party in private firms promotes CSR performance, but this is influenced by the political connections of the firm owners. In addition to providing empirical support for the study of corporate behavior in the Chinese context, this paper identifies the impact and development trajectory of the party branches of China’s ruling party on CSR. However, this paper does not discuss the implicit costs required for party branches to promote CSR, and we hope that future research will make further explorations.

## 1. Introduction

In a policy statement for private businesses published in September 2020, the Central Committee of the Communist Party of China emphasized the necessity of “consolidating and broadening political consensus, further strengthening the party building work of private enterprises, and effectively playing the role of the party organization as a fighting fortress and the vanguard and exemplary role of party members.” Party branches embedded in private businesses are now a very widespread political and economic phenomena in China since they are a legal institutional structure that is explicitly outlined in the *Articles of the Communist Party of China* and the *Company Law of the People’s Republic of China*. According to the “Intra-Party Statistical Bulletin of the Communist Party of China” issued by the Central Committee of the Communist Party of China, as of June 5, 2021, 1.513 million grass-roots party organizations have been established in firms in mainland China, and the embeddedness of party branches and corporate governance have been deeply integrated. Has the Communist Party’s widespread and broad engagement in businesses have the anticipated political and economic effects?

The effect of party branches operating inside of businesses has been studied by several academics. Enhanced party building may greatly increase corporate governance in Chinese SOEs, according to Bi (2021) and Yu et al. (2021), on the other hand, find that acquisitions with their own party committees create significantly higher market value for acquiring firms. Additionally, Li and Cheng (2021) discover that from the standpoint of R&D and innovation, the engagement of Communist Party branches in the activities of Chinese listed enterprises decreases innovative inputs but enhances firms’ innovation performance. As is evident, the majority of past studies have employed state-owned businesses as their study sample. But the different nature and lifecycle of enterprises can lead them to behave differently (Durana et al., 2021). While for private enterprises, political ties to the Communist Party have been discovered to aid enterprises access external resources, as seen in the case of the Chinese private firm’s debt financing and business performance (Haveman et al., 2017; Valaskova et al., 2021). Additionally, this relationship is related to regional institutional environment and industrial policy (Zhang et al., 2022).

Chinese firms are exposed to political influence from three primary sources: state shareholding, government and ministries, and party committees. Political influence from government and state-owned shareholders affects corporate behavior primarily by influencing the allocation of external resources. However, the party branch is directly embedded in the internal organization and governance structure of the company and intervents in a more direct way. Although China’s political system is based on “party-government unity,” from the perspective of the “administrative-political” dichotomy, due to the principal-agent relationship and institutional inertia, the government and state-owned shareholders may also produce strategic administrative phenomena like “political tournaments” at the local grassroots level and at the individual enterprise level, deviating from the wishes of political parties (Feldman et al., 2021). Additionally, due to the special institutional environment in China, companies sometimes give up some economic benefits in order to obtain other resources. Thus, rather of concentrating simply on commercial concerns, political engagement from the corporate party branch might affect the enterprise’s non-profit activities. Zhang et al. (2021) found that corporate party committee governance enhanced corporate willingness to donate in a study of Chinese listed firms in the heavy-pollution industry. Studies have also found that Communist Party interventions, such as corporate party branches and inculcation of CEO ideology, promote environmental corporate social responsibility (ECSR) among Chinese firms (Zhou et al., 2021; Su and Zhang, 2022). As noted above, research that have hitherto been conducted on the impact of corporate party branches on nonprofit activity have mostly emphasized corporate social responsibility in a wide sense.

Corporate social responsibility (CSR) is a broad concept that can take many forms. Businesses can benefit society while boosting their brands through CSR programs such as philanthropy and volunteer efforts. Therefore, exploring the factors affecting CSR has long been an important topic of interest for scholars. Previous studies have analyzed the factors affecting CSR from several perspectives, such as tournament incentives (Khan et al., 2022) and non-financial disclosure (Jackson et al., 2020), but few studies have focused on the impact of political intervention of the ruling party on CSR. In particular, there was a lack of discussion of the mechanisms of influence. According to the social embeddedness theory, social interactions and social network structure have an impact on economic behavior through affecting a number of mediating elements, including resource availability, emotional support, and knowledge sharing (Granovetter, 2018). Party branches that are embedded in the firm also play a “supervisory” and “leadership” function, affecting the way the business acts. Private firms in China are typically less perceptive of policy than state-owned firms with links to the institution. According to signaling theory, in order to reduce of information asymmetry, information must be sent across organizations *via* certain signals. By embedding into the governing structure of private enterprises, the party branch transmits the CCP Central Committee’s will, enhances the perception of policies among private enterprises, and influences their behavior by integrating them into the party organization network across China. This article aims to investigate whether party branches impact CSR performance through improving the private enterprises’ policy perception.

This study investigates the impact of the party branch as a kind of formal institutional structure on the social responsibility of private firms based on the 11th Chinese Private Enterprise Survey (CPES). This article also aims to investigate the mediation effect of policy perception ability in order to more precisely identify its effect path. We found that, first, the embedding of party branches will improve the CSR of private enterprises in different dimensions, by enhancing the policy perception of private enterprises. Second, in various influence pathways, the party branches will enhance the perceptions of policy related to economic interests, which has a more significant impact on enhancing the performance of philanthropic CSR. Finally, by examining the effects of moderation, it can be shown that business owners’ first-given and later-generated political connections support the party branches’ perception of policies related to economic and social interests, respectively. After adjusting for endogeneity using propensity score matching, and Heckman two-step method, these results remain valid.

The theoretical contributions of this study in comparison to earlier research are as follows. First, by using Chinese private firms as the research subject, this work broadens the research viewpoint on the impact of Communist Party branches on corporate conduct and enhances the pertinent research literature. Second, the majority of current research on CSR is undertaken from a single perspective, and only a small number of empirical evaluations are done from a multidimensional perspective. It is inevitable that managers’ initial motivations would differ, which will result in different CSR performance. The varied influences of party branches on various forms of CSR are thoroughly examined in this work, which also advances the body of knowledge about the effects of party branches on corporate governance. Third, this research focuses on the mediating effect of policy perceptions based on social embeddedness theory and signaling theory. The influence paths of party branches in private businesses are explained by the empirical evidence presented in this paper.

## 2. Literature review and hypotheses

### 2.1. Concepts and dimensionality of corporate social responsibility

The term “corporate social responsibility” (CSR) refers to what society expects from a business organization. It calls for businesses to look beyond conventional corporate objectives like “profit first” and place an emphasis on contributions to all stakeholders, such as the employees, clients, and society. According to the stakeholder theory, an organization’s business risks are shared by all of its stakeholders, hence it should adopt social responsibility as a risk mitigation strategy (Dmytriyev et al., 2021). Because it provides a more comprehensive response to the question of “to whom should corporations be accountable,” it has emerged as the dominant theory in the field of CSR research. The classic CSR pyramid model treats CSR as a structural component, incorporating economic, legal, ethical, and philanthropic duties from the bottom up. Environmental, Social and Governance (ESG) has received a great deal of attention in recent years and has influenced the way researchers view CSR, with an increasing focus on the environmental dimension of CSR (Gillan et al., 2021). However, most of studies still categorize CSR as ranging from economic to philanthropic.

The existing research reveals that there are several motivations for businesses to fulfill CSR, including the four main points listed below. First, there is the altruistic motivation for altruism, which views CSR as a physical manifestation of ethical behavior (Miller et al., 2022). Second, the strategic motivation, which uses CSR to support the company’s growth strategy (Nave and Ferreira, 2019). By carrying out its social obligation, the business may demonstrate to the outside world that it works responsibly, earning the respect of external stakeholders and enhancing its reputation and legitimacy. Third, the managerial self-interested motivation to achieve other specific goals, which is essentially a self-interested behavior, such as the enterprise hopes that through good social responsibility performance as a crisis and impression management tool, thus improving the company’s reputation and public image (Du, 2015). Fourth, political incentive, businesses must do well in CSR to satisfy political demands (Xu and Liu, 2020). Chinese businesses are driven more by political and self-interest to fulfill their social obligation. Many businesses desire to leverage their strong CSR performance to further economic and political objectives, such as reducing the risk of litigation’s erosion of credibility and legitimacy (Du, 2015). Therefore, its performance in many dimensions may depending on the different motivations. We contend that a multifaceted analysis of the specific performance of CSR is required.

### 2.2. Social embeddedness, signaling and mediation effects

Chinese private businesses are integrated into the Communist Party of China’s organizational network *via* party branches located inside such businesses. According to Granovetter (2018), organizations must construct information channels with the strength of weak ties if they wish to retain effective ties. Party branches serve as an important channel of communication and contact with Chinese authorities, despite the fact that they are typically not directly involved in the management of private enterprises. Yan and Xu (2022) found that the Chinese Communist Party branch as a crucial communication bridge between the government and firms, which transfers public governance goals to private enterprises, and promotes employment protection. They also integrate private businesses into the organizational network of the CCP by acting as a kind of interorganizational social contact. He and Liu (2022) found that Chinese authorities exert political pressure on private enterprises to set up party branches within the firm intending to regain political influence, and this pressure forces family firms to comply with the requirement to set up party branches and thus build an organizational network of the party.

According to signaling theory, sending signals across organizations helps reduce information asymmetry. Chinese private businesses frequently lack connections to the system, in contrast to state-owned companies’ inherent political links (Wang et al., 2022). As a result, when the CCP communicates private businesses of its policy intentions, there are frequently weak channels and poor execution. By arranging actions for party building, the party branches embedded in private businesses objectively perform the function of sending signals (He and Liu, 2022). Party branches act as “supervision” and “leadership” entities by promptly communicating the most recent policy intent to businesses, so reducing the problem of information asymmetry. However, very few research have been done to investigate the mechanisms behind the influence of the party branch. Party branches often do not participate directly in the administration of private businesses; instead, they influence and control businesses through indirect channels such as political propaganda (Mittelstaedt, 2021). Because of this, it is important to take into account the relevant mediating effects in order to understand the function and impact mechanism of the party branch embedded in private enterprises.

### 2.3. Institutional context and research hypothesis

The economic system in mainland China has changed a lot in recent years. Prior to the “reform and opening up,” private capital was rarely allowed to engage in economic activity during the planned economy era. As a result, economic development had to be handled by state-owned enterprises. Prior to the reform and opening up, state-owned firms in China engaged into implicit long-term labor contracts in the form of unit ownership with their employees and assumed various public benefits, such as employment, health care, pensions, housing, and education. And they carried out their production under the coordination of a command planning economy. At this stage, however, businesses are offering more social benefits than they can reasonably afford, which has had detrimental effects including low productivity and free-riding (Cai, 2019).

The 11th Central Committee of the Communist Party of China’s Third Plenary Session brought about a fundamental change in the country’s economic system. China’s economic system underwent a phase of transition from a planned economy to “socialist market economy,” in which private firms were permitted to function and state-owned enterprises progressively withdrew from the majority of economic sectors. China’s economic reform, however, has led to “big layoffs” and the serious loss of state-owned assets as a result of poor management. The rights of the environment, employees, and customers have also been violated by the brutal expansion of many private enterprises. Even some people have expressed the opinion that these private enterprises may have original sin (Shi et al., 2022). In general, Chinese private businesses at this time did not take corporate social responsibility very seriously, which had major negative externalities. Enterprises are regulated and compelled to pay attention to and take responsibility for CSR as China’s market opens up and its legal system steadily improves. Private companies in China are also fulfilling CSR by enhancing working conditions and making charity donations (Lau et al., 2016).

Private businesses in China are required to create party branch committees. Do party branches that are embedded in businesses, which are a crucial source of knowledge about policy for company owners, have an impact on how well CSR is carried out in private enterprises? The Constitution of the Communist Party of China defines the “supervision” and “leadership” roles that party branches play in private businesses. Party branches influence private enterprises to respond to the Party’s political views through semi-obligatory propaganda, so enhancing the enterprises’ feeling of social responsibility (Dong et al., 2016). According to Gupta et al. (2017), ideologies that come from organizations are more likely to support CSR than ideologies that come from individuals. The private enterprises embedded in the party branch are connected to the organizational network of the Communist Party and develop a unified organizational perspective under the “supervision” and “leadership” of the party branch (Yan and Xu, 2022). As a result, they may be affected by the party’s wishes and invest heavily in CSR than other businesses. Therefore, we propose the following research hypothesis.

*H1*: The Party Branch will promote the fulfillment of CSR in Chinese private enterprises.

Party branches often do not participate directly in the business administration of Chinese private firms, in contrast to state-owned ones. Instead, they have an indirect impact on corporate behavior by altering the internal informational environment, and this effect alters how business owners view the external political environment (Yan and Xu, 2022). According to the social cognitive theory of triadic reciprocal causation, the environment can impact conduct *via* altering perception (Bergman et al., 2019). Different perceptions of business owners can lead to different decisions. For a long time, Chinese private enterprises have had poor policy perception abilities, and created significant governance challenges (Dai and Si, 2018). However, as China’s economic reform moves forward, this circumstance is progressively getting better. According to Article 19 of The People’s Republic of China’s Company Law: “In companies, the organization of the Communist Party of China shall be established and its activities shall be carried out in accordance with the provisions of the Constitution of the Communist Party of China. The company shall provide the necessary conditions for the activities of the Party organization.” The embedding of party branches in private enterprises is formally institutionalized under the legislation. The Communist Party of China’s Constitution stipulates that the party branch of private enterprises is responsible for monitoring business operations through propaganda. Through events like party congresses, it transfers the Party Central Committee’s policy spirit to the internal workings of the company, enhancing private enterprises’ capacity for policy perception. The capacity to perceive policy better will alter private enterprise goals through mediating effect, forcing enterprises to invest significant resources in CSR for cater to the Party values. As a result, we propose the following research hypothesis.

*H2*: The Party Branch will promote policy perception in Chinese private enterprises.

*H3*: Policy perception mediates the relationship between party branch and CSR performance.

## 3. Methodology

### 3.1. Sample and data selection

This study is devoted to mapping party branches, policy perceptions and CSR, and uses Chinese small and medium-sized private enterprises as a research sample. This requires important business data about the firms, as well as information about the entrepreneurs’ political identities. In China, it is difficult for academic institutions to obtain this information through independent investigation, which requires the assistance of official institutions. The 11th China Private Enterprise Survey (CPES) in 2014 provided the sample data used in this study. The survey was carried out under the auspices of a committee that was jointly established by the State Administration for Industry and Commerce of China, the Federation of Industry and Commerce, the United Front Work Department of the CCP Central Committee, and other authority departments. A sample of private enterprises with excellent representativeness and reliability is created by using a multi-stage sampling procedure. It includes company information and indicators of the personal characteristics of business owners, covers a total of 6,144 private firms across 31 provincial-level units and 19 industry categories in mainland China, and has good data quality and sample representativeness. Samples that lacked important factors were eliminated from this study before the analysis began. Additionally, all continuous variables were winsorized at the 1% level in order to remove the impact of outliers. Finally, 5,005 valid cross-sectional data samples were acquired.

The establishment of party branches in private enterprises is a unique political and economic phenomenon in China. Because this nation has maintained a one-party dictatorship despite implementing market-oriented reforms, and only a small number of nations fit this requirement. Although most multi-party democracies lack the necessary conditions to establish party branches in private enterprises, it is nevertheless possible to replicate the research in different conditions. In terms of methodological steps, we suggest that researchers in other countries first consider how to gather trustworthy data on firms’ business and political connections and then focus on concretely defining the vague concept of policy perception. For instance, researchers can use corporate political donations to gauge the effect that party affiliation has on them. The impact of this on the entrepreneur’s perception of the party’s policies is next examined, followed by a look at whether it has an impact on the business’s strategy.

### 3.2. Measurement

#### 3.2.1. Dependent variables

Corporate Social Responsibility ( $CSR_{i}$ ). The concept of the pyramid model of CSR is used in this study to quantify the social responsibility of businesses in several dimensions by focusing on the performance of three private firm areas: labor protection, environmental protection, and charity giving. Among them, CSR of labor ( $CSR_{1}$ ) and CSR of environmental ( $CSR_{2}$ ) are economic and strategic, while CSR of charitable ( $CSR_{3}$ ) is more altruistic and philanthropic. We use the amount of employee training and social insurance paid by the company to measure $CSR_{1}$ ; the amount of environmental protection paid by the company to measure $CSR_{2}$ ; and the amount of donation to charity to measure $CSR_{3}$ . For each of the aforementioned variables, a logarithm is applied.

#### 3.2.2. Independent variables

Party branch ( CCP ). To identify if the party branch was embedded into the firm, we generated dummy variables and utilized the CCP organization’s establishment in a private firm as a proxy variable. If a party organization was established in the sample firms, CCP was given the value 1, otherwise it was given the value 0.

#### 3.2.3. Control variables

Firm characteristics ( Firm ) and owner characteristics ( Owner ) were chosen as control variables in relation to the dependent variable. The following variables serve as controls with regard to firm characteristics: firm age ( Coage ), operational income ( Income ), net profit ( Profit ), firm size ( Size ), and board of directors ( Board ). The following variables serve as controls with regard to owner characteristics: Gender ( Gender ), Age ( Age ), Education ( Education ), and Political Identity ( PI ). In our research, we also control for the fixed effects of industry and province. The definitions of the variables are displayed in Table 1.

**Table 1:** Variable definitions.

Variable type		Variable name	Symbol	Definition
Dependent variables		CSR of labor	$CSR_{1}$	Employer-paid social insurance premiums and employee training costs, totaled and take the logarithm
		CSR of environmental	$CSR_{2}$	The environmental pollution control costs by enterprises, take the logarithm
		CSR of charitable	$CSR_{3}$	The amount of corporate donations to charitable causes, take the logarithm
Independent variables		Party branch	CCP	Whether the enterprise established a party branch (Yes = 1, No = 0)
Intermediate variables		Policy perception	Perception	Business owners’ perception of and participation in the six policies and activities (“better understand, participated in” is assigned a value of 2; “heard, some understanding” is assigned a value of 1; “do not know” is assigned a value of 0, the same below)
		Policy perception for profit	Tool	Business owners’ perception of and participation in three policies and activities that are closely linked to the economic interests of the business
		Nonprofit policy perception	Value	Business owners’ perception of and participation in three policies and activities that are closely linked to social interests
Control variables	Firm characteristics ( Firm )	Firm age	Coage	Statistical year – year of business registration
		Operational income	Income	Total operating income, normalized
		Net profit	Profit	The amount of net profit, normalized
		Firm size	Size	Total number of employees, take the logarithm
		Board of directors	Board	Whether or not the company has a board of directors (Yes = 1, No = 0)
	Owner characteristics ( Owner )	Gender	Gender	Gender of business owner (male = 1, female = 0)
		Age	Age	Statistical year - year of birth of the business owner
		Education	Education	Whether or not the business owner has Whether or not the business owner has university study experience (Yes = 1, No = 0)
		Political identity	PI	Whether or not the business owner is serving as a delegate to the People’s Congress (PC) or a member of the Political Consultative Conference (PCC) (Yes = 1, No = 0)

### 3.3. Statistical method

**Figure 1:**
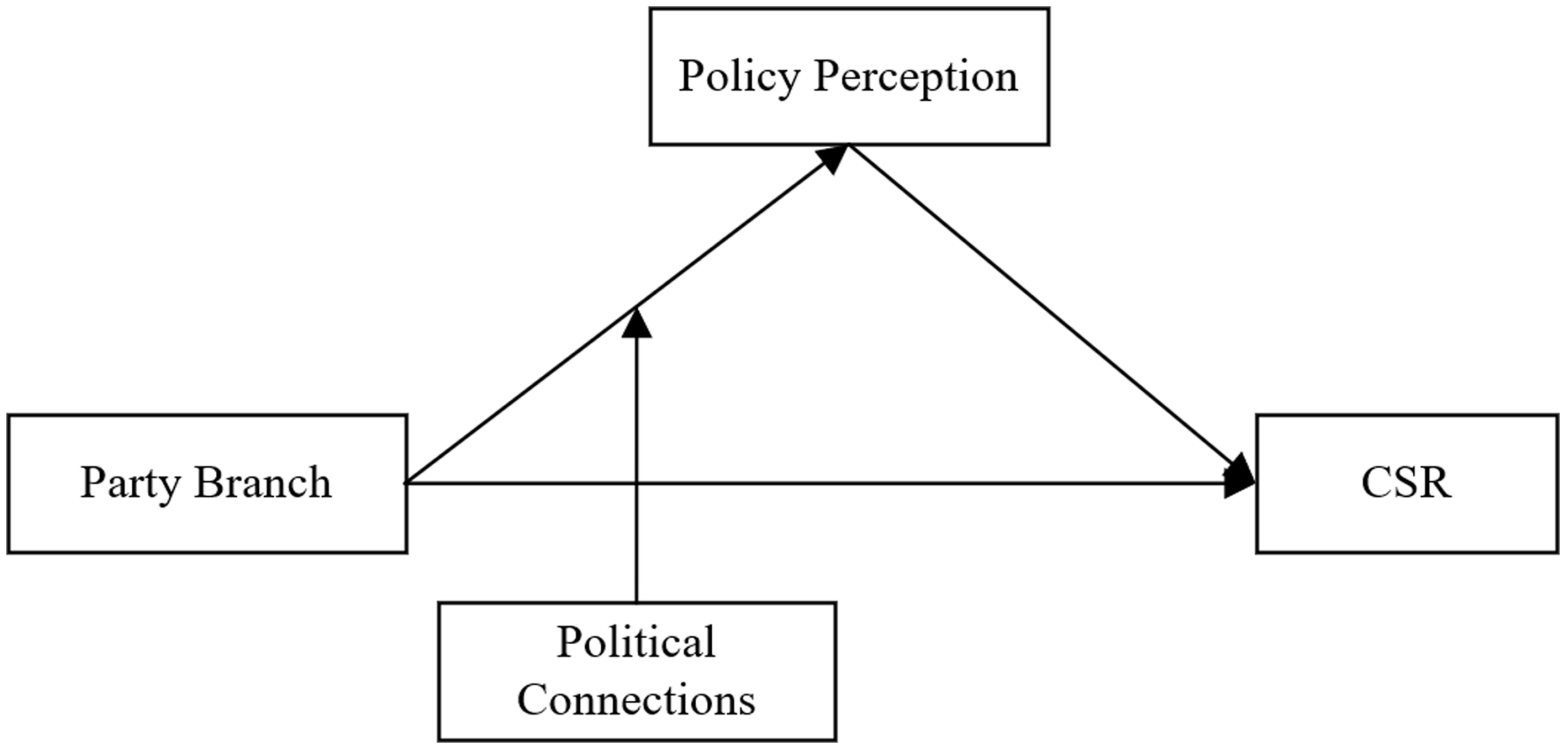
Conceptual model.

In this study, we used ordinary least squares (OLS) regression to serve as the baseline regression, and a fixed effects model is used in the baseline regression so as to address endogeneity. The following econometric model was developed to investigate whether party branches improve CSR in Chinese private enterprises.


(1) $$CSR_{i}=\alpha_{0}+\beta_{i}CCP+\gamma_{1}Firm+\gamma_{2}Owner+\eta_{j}+\lambda_{k}+\varepsilon_{i}$$

where $CSR_{i}$ is three dimensions of CSR ( i =1,2,3), Firm and Owner are control variables for firm characteristics and owner characteristics, respectively. $\eta_{j}$ and $\lambda_{k}$ denote province and industry fixed effects, and $\varepsilon_{i}$ is a random error term. This paper will also use a variety of models to test for fixed effects, such as the causal steps approach, the Soble test, and the Bootstrap structural equation model (SEM) to make the results more credible. On this basis, moderating effects of political connections will also be considered. To sum up, the conceptual model is displayed in Figure 1.

## 4. Analysis and results

### 4.1. Descriptive analysis

Table 2 presents descriptive statistics for the primary variables. The findings show that the mean value of CCP is 0.370, meaning that 37% of the sample firms had a party branch in place. In terms of firm characteristics, the mean value of Coage is 10.832, indicating that Chinese private businesses are often new. This may be due to the late opening up of China and the short average lifespan of private enterprises. And the operating circumstances of Chinese private enterprises are severely polarized, as evidenced by the mean value of the normalized operational income being negative. In particular, the average of the raw data on corporate net profits is also negative, showing that Chinese private enterprises have a typically bad chance of surviving in business.

**Table 2:** Descriptive statistics of the variables.

	Number	Average	Standard deviation	Minimum	Maximum
CCP	5,005	0.370	0.483	0.000	1.000
$CSR_{1}$	5,005	9.840	5.349	0.000	16.831
$CSR_{2}$	5,005	4.865	5.973	0.000	16.524
$CSR_{3}$	5,005	7.298	5.542	0.000	15.568
Coage	5,005	10.832	6.033	1.000	27.000
Income	5,005	−0.032	0.109	−0.067	0.768
Profit	5,005	0.012	0.032	−0.009	0.231
Size	5,005	4.119	1.594	1.099	8.033
Board	5,005	0.575	0.494	0.000	1.000
Gender	5,005	0.851	0.357	0.000	1.000
Age	5,005	45.431	8.450	25.000	64.000
Education	5,005	0.369	0.483	0.000	1.000
PI	5,005	0.366	0.482	0.000	1.000

As for the characteristics of business owners, the mean value of Gender is 0.851, and the proportion of male business owners is much higher than that of female. The average age was 45.431, with the youngest being 25 years old and the oldest being 64 years old. The mean value of PI is 0.366 shows that 36.6% of business owners are now NPC deputies or CPPCC members, indicating that Chinese private business owners are more engaged in politics. We tested the variance inflation factor before the regression analysis. With a maximum VIF value of 1.77, serious multicollinearity was not seen.

### 4.2. Regression analysis and results

Table 3 shows the model’s OLS regression findings, where columns (1)–(3) control for firm characteristics only, while columns (4)–(6) control for business owner characteristics additionally. The regression findings demonstrate that, whether in the areas of labor protection, environmental protection, or charity giving, the Party branch greatly enhances the CSR performance of Chinese private enterprises. When business owner characteristics were not taken into account, the estimated party branch coefficients on CSR were 0.918, 1.376, and 1.574, all of which were significant at the 1% level. The coefficients are 0.799, 1.338, and 1.261, respectively, after taking account for the business owner characteristics, and they are also significant at the 1% level. H1 is therefore proved.

**Table 3:** Party branches and CSR in Chinese private enterprises: OLS regression.

	(1)	(2)	(3)	(4)	(5)	(6)
	$CSR_{1}$	$CSR_{2}$	$CSR_{3}$	$CSR_{1}$	$CSR_{2}$	$CSR_{3}$
CCP	0.918^***^	1.376^***^	1.574^***^	0.799^***^	1.338^***^	1.261^***^
	(0.160)	(0.178)	(0.167)	(0.162)	(0.180)	(0.167)
Coage	0.036^***^	0.007	0.125^***^	0.029^**^	0.005	0.107^***^
	(0.012)	(0.013)	(0.013)	(0.013)	(0.014)	(0.013)
Income	−0.454	2.036^**^	−0.125	−0.726	2.134^**^	−0.348
	(0.867)	(0.962)	(0.902)	(0.866)	(0.963)	(0.890)
Profit	1.237	−0.234	7.651^**^	0.960	−0.123	7.414^**^
	(2.906)	(3.223)	(3.022)	(2.898)	(3.221)	(2.977)
Size	1.275^***^	1.092^***^	1.071^***^	1.207^***^	1.086^***^	0.933^***^
	(0.057)	(0.063)	(0.059)	(0.058)	(0.065)	(0.060)
Board	0.004	0.011	−0.355^**^	−0.034	0.042	−0.349^**^
	(0.136)	(0.151)	(0.141)	(0.136)	(0.151)	(0.140)
Gender				−0.073	0.176	0.040
				(0.190)	(0.211)	(0.195)
Age				0.016^*^	−0.009	0.002
				(0.009)	(0.010)	(0.009)
Education				0.736^***^	−0.489^***^	0.042
				(0.149)	(0.165)	(0.153)
PI				0.399^***^	0.444^***^	1.949^***^
				(0.152)	(0.169)	(0.156)
Industry	Yes					
Province	Yes					
Constant	0.429	−1.878^***^	−1.607^***^	−0.137	−1.625^**^	−1.642^**^
	(0.534)	(0.592)	(0.555)	(0.639)	(0.709)	(0.656)
*N*	5,005	5,005	5,005	5,005	5,005	5,005
*R*-squared	0.252	0.262	0.246	0.257	0.264	0.270

^***^p < 0.01, ^**^p < 0.05, ^*^p < 0.1; standard errors in parentheses, same as in the following tables.

Our main worry is the sample’s bias due to selection, which makes it difficult to determine whether party branches influence the CSR performance of Chinese private enterprises. That is, rather than being randomly selected, party branches embedded in private enterprises are more likely to be found in larger companies. Large companies may perform better in CSR than smaller ones, which introduces systematic bias and makes it challenging to identify causal relationships. As a result, extra tests are carried out in this research based on model (1), where CSR expenditures are adjusted for the number of employees. The results are broadly in line with Table 3, and the coefficients that we are interested in are all positively significant at the 1% level, suggesting that the influence of sample bias may not be as significant as it may first estimate.

### 4.3. Mediating effects of policy perception

The previous regression results show that the party branch significantly enhances the CSR performance of Chinese private enterprises. What thus is the party branch’s mechanism for promoting CSR in private enterprises? We will examine the mediating role that private firms’ policy perceptions play in order to address this problem. Specifically, we assigned values to enterprises’ perceptions of and engaged in the six policies and activities: “*Opinions on Further Supporting the Healthy Development of Small and Micro Enterprises*” (Article 29 for small and micro enterprises), “*Several Opinions of the State Council on Encouraging and Guiding the Healthy Development of Private Investment*” (Article 36 for private investment), “*Guiding Opinions on Financial Support for Economic Restructuring and Transformation and Upgrading*,” “For the People, Pragmatic and Clean” Party’s Mass Line Education and Practice Activities, the Guangcai Project, and “Private Entrepreneurs and the Chinese Dream” education and practice for people in the non-public sector. The policy perception variable is assigned a value of 2 for “know, attended,” 1 for “heard, know,” and 0 for “do not know.” Use Perception as a proxy variable with a maximum value of 12 and a minimum value of 0 to determine how private enterprises are perceived in terms of policy.

We initially carry out a mediating effects test utilizing the causal steps approach. The model is setup as shown below.


(2) $$CSR_{i}=\alpha_{0}+\beta_{i}CCP+\gamma_{1}Controls+\eta_{j}+\lambda_{k}+\varepsilon_{i}$$

(3) $$Perception=\alpha_{0}+\beta_{i}CCP+\gamma_{1}Controls+\eta_{j}+\lambda_{k}+\varepsilon_{i}$$

(4) $$CSR_{i}=\alpha_{0}+\beta_{i}CCP+\delta_{i}Perception+\gamma_{1}Controls+\eta_{j}+\lambda_{k}+\varepsilon_{i}$$

The coefficients $\beta_{i}$ and $\delta_{i}$ in Equations 3, 4 should both be positively significant if policy perception mediates the relationship between the party branch and CSR. Additionally, after controlling for Perception , the coefficient βi' s value in Equation 4 should drop or lose significance in comparison to the value in Equation 2. The findings of the mediating effects test are presented in Table 4. According to the results in column (1), the coefficient $\beta_{i}$ in Equation 3s has an estimated value of 0.901, which is positively significant at the 1% level. The regression coefficients of Perception on $CSR_{i}$ in columns (3), (5) and (7) are 0.089, 0.206 and 0.391, respectively, all of which are positively significant at the 1% level. The results in columns (2)–(7) show that the coefficients of $CSR_{i}$ all decrease after controlling for policy perception ( $CSR_{1}$ decreases from 0.799 to 0.718; $CSR_{2}$ decreases from 1.338 to 1.153; $CSR_{3}$ decreases from 1.261 to 0.909). The aforementioned results suggest that the party branch may improve CSR performance by improving the policy perception of the owner of private enterprises. Thus, H2 and H3 are verified.

**Table 4:** Party branches and CSR in Chinese private enterprises: Mediating effects of policy perception.

	(1)	(2)	(3)	(4)	(5)	(6)	(7)
	Perception	$CSR_{1}$	$CSR_{1}$	$CSR_{2}$	$CSR_{2}$	$CSR_{3}$	$CSR_{3}$
CCP	0.901^***^	0.799^***^	0.718^***^	1.338^***^	1.153^***^	1.261^***^	0.909^***^
	(0.087)	(0.162)	(0.164)	(0.180)	(0.181)	(0.167)	(0.165)
Perception			0.089^***^		0.206^***^		0.391^***^
			(0.026)		(0.029)		(0.027)
Coage	0.050^***^	0.029^**^	0.024^*^	0.005	−0.006	0.107^***^	0.087^***^
	(0.007)	(0.013)	(0.013)	(0.014)	(0.014)	(0.013)	(0.013)
Income	0.272	−0.726	−0.751	2.134^**^	2.078^**^	−0.348	−0.455
	(0.465)	(0.866)	(0.866)	(0.963)	(0.958)	(0.890)	(0.871)
Profit	2.501	0.960	0.736	−0.123	−0.638	7.414^**^	6.436^**^
	(1.554)	(2.898)	(2.896)	(3.221)	(3.206)	(2.977)	(2.916)
Size	0.313^***^	1.207^***^	1.179^***^	1.086^***^	1.022^***^	0.933^***^	0.810^***^
	(0.031)	(0.058)	(0.059)	(0.065)	(0.065)	(0.060)	(0.059)
Board	−0.126^*^	−0.034	−0.022	0.042	0.068	−0.349^**^	−0.300^**^
	(0.073)	(0.136)	(0.136)	(0.151)	(0.150)	(0.140)	(0.137)
Gender	−0.112	−0.073	−0.063	0.176	0.199	0.040	0.084
	(0.102)	(0.190)	(0.190)	(0.211)	(0.210)	(0.195)	(0.191)
Age	0.014^***^	0.016^*^	0.015	−0.009	−0.012	0.002	−0.003
	(0.005)	(0.009)	(0.009)	(0.010)	(0.010)	(0.009)	(0.009)
Education	0.228^***^	0.736^***^	0.716^***^	−0.489^***^	−0.536^***^	0.042	−0.047
	(0.080)	(0.149)	(0.149)	(0.165)	(0.165)	(0.153)	(0.150)
PI	1.117^***^	0.399^***^	0.299^*^	0.444^***^	0.214	1.949^***^	1.512^***^
	(0.081)	(0.152)	(0.155)	(0.169)	(0.171)	(0.156)	(0.156)
Industry	Yes						
Province	Yes						
Constant	3.147^***^	−0.137	−0.419	−1.625^**^	−2.273^***^	−1.642^**^	−2.873^***^
	(0.342)	(0.639)	(0.643)	(0.709)	(0.712)	(0.656)	(0.648)
*N*	5,005	5,005	5,005	5,005	5,005	5,005	5,005
*R*-squared	0.255	0.257	0.259	0.264	0.272	0.270	0.300

^***^p < 0.01, ^**^p < 0.05, ^*^p < 0.1.

However, there is a query as to what is the purpose of Chinese private enterprises to perceive policies? According to institutional theory, on the one hand, private enterprises can enhance their awareness of the institutional environment in order to better understand changes in policies, adjust their business plans, and enhance their economic performance. On the other hand, institutional pressure can improve CSR performance through community isomorphism (Roszkowska-Menkes et al., 2017). Strategic choice theory suggests that a manager’s perception of the external environment is the primary element determining a company’s strategic decisions. Varied ways that business owners perceive political climate will ultimately result in different strategies that businesses behave. We thus think it is necessary to investigate if there are differences in the impact effects of party branches on different policy perceptions, and then on the CSR performance of private enterprises.

We subsequently investigated enterprises’ perceptions of policy related to economic interests ( Tool ) and those related to social interests ( Value ) based on the analyses mentioned above. We categorize the problems that make up Perception in detail. The same as previously, we assign values to three policies that are directly connected to economic interests: “Article 29 for small and micro enterprises,” “Article 36 for private investment,” and “Guidance Opinions.” Then, the variable Tool , with a maximum value of 6 and a minimum value of 0, is used as a proxy variable to measure the degree of private enterprises’ perceptions of policy related to economic interests. At the same time, the enterprises’ participation in the “Party’s Mass Line Education and Practice Activity,” “Guangcai Project,” and “Education and Practice of Non-public Economic Personnel” was assigned a value, and the same is treated as a variable Value with a maximum value of 6 and a minimum value of 0 as a proxy variable for the degree of private enterprises’ perceptions of policy related to social interests.

We use the Soble test instead of the causal steps approach in the next analysis to quantify the mediating effect. In addition, we also used the KHB test, which loosens the linear model’s presumptions. Both approaches enable the estimation of the proportion of mediating effects. The results of Sobel test and KHB test are reported in Table 5. According to the results, Tool and Value are significantly important mediating factors in each path. In particular, the mediating effect that Party Branch enhance CSR of charitable ( $CSR_{3}$ ) by enhancing the perceptions of policy related to social interests ( Value ) of Chinese private enterprises is more obvious. The proportion of party branches increasing $CSR_{3}$ through this effect achieved 33.16% and 31.01%, respectively, according to the results of the Sobel test and the KHB test. In general, party branches have a more important role in boosting corporate social responsibility of charitable by improving the perception of policies related to social interests.

**Table 5:** Heterogeneity policy perceptions: the Sobel & KHB test.

Independent variable	Intermediate variables	Dependent variable	Sobel test				KHB test	
			Direct effect	Indirect effects	Total effect	Share of intermediary effect	Intermediary Effect	Share of intermediary effect
CCP	Tool	$CSR_{1}$	0.912^***^	0.032^**^	0.944^***^	3.42%	0.019	2.03%
		$CSR_{2}$	1.531^***^	0.077^***^	1.608^***^	4.77%	0.047^**^	2.93%
		$CSR_{3}$	1.119^***^	0.133^***^	1.252^***^	10.64%	0.045^**^	3.56%
	Value	$CSR_{1}$	0.875^***^	0.069^***^	0.944^***^	7.30%	0.057^**^	6.08%
		$CSR_{2}$	1.449^***^	0.158^***^	1.608^***^	9.85%	0.130^***^	8.08%
		$CSR_{3}$	0.837^***^	0.415^***^	1.252^***^	33.16%	0.388^***^	31.01%

^***^p < 0.01, ^**^p < 0.05.

**Figure 2:**
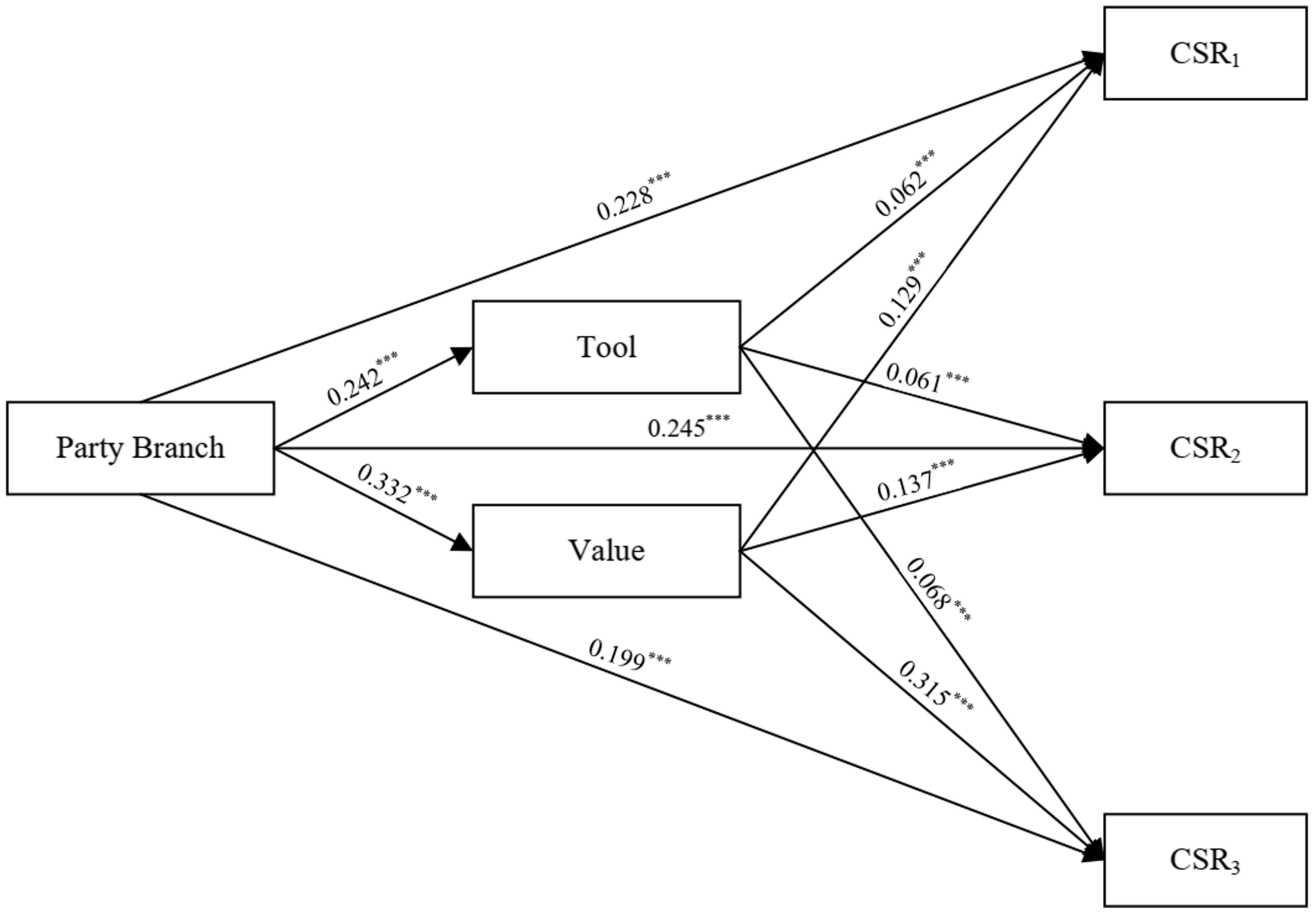
Structural equation model path analysis. ***p < 0.01.

According to Wooldridge (2010), the normality assumption must be met by the sampling distribution of the mediating variables in order for the Sobel test to be valid. Therefore, as an alternative, we use the Bootstrap structural equation model, which does not depend for such assumption of normality. The mediating effect test of SEM, which is depicted in Figure 2, confirms the plausibility of the findings and is consistent with the previous findings.

### 4.4. Moderating effects of political connections

According to Tijani et al. (2021), a strategic alliance and partnership is an important tool for small private enterprises’ survival. But in post-communist countries such as China, political connections are also important for the survival of enterprises. Chinese private enterprises are more interested in developing political relationships to obtain government resource support as contrasted to the inherent political advantages that state-owned enterprises enjoy (Zhang et al., 2022). In addition to the political connections that private enterprises establish for the purpose of business interests, Chinese authorities also want to use their political connections to influence private enterprises. It is possible to distinguish between “first-given political connections” and “later-generated political connections” based on the time of business owners’ acquisition of these relationships. The distinction between the two is whether the political connections were made before or after the business owner founded the enterprise. The government positions held by the business owner before to founding the business can be used to gauge the “first-given political connections.” With access to this institutional resource, business owners may develop a cognitive imprint and improves their capacity to recognize policy possibilities. “Later-generated political connections” often refer to the political status that a business owner has attained after founding their business, such as current membership in the National People’s Congress, or the Chinese People’s Political Consultative Conference (Fan et al., 2007; Zhang et al., 2016). In addition, Chinese private enterprises also develop political connections by hiring former government officials (Cheng, 2018).

Therefore, we investigate the moderating effects of both the “first-given” and “later-generated” political connections on the perceptions of policy held by private enterprises. Denote “first-given political connections” by institutional capital ( IC , if the business owner held a government leadership position before establishing the firm) and “later-generated political connections” by party official ( PO , if the business owner held a position in the Communist Party). The results of the test for moderating effects are shown in Table 6. The exact test results of the control variables are not reported later due to space constraints. In columns (2), (3), (5), and (6), the estimated coefficients of the interaction terms CCP×IC and CCP×PO are all positive. Additionally, there are significant effects for both the influence of PO on Value and the effect of IC on Tool . This result is generally consistent with the findings of Chen and Cao (2016). It’s possible that this is the case because business owners who formerly held leadership positions in bureaucracy are better versed with the Chinese government’s political subterfuges and have a broader network of contacts. This group of business owners left their influential government jobs to pursue their businesses, placing a greater focus on using political ties for financial advantage. Rich Chinese private business owners, however, do not yet have the commensurate social standing; as a result, they choose to utilize their economic might to forge political relationships and rise in social standing.

**Table 6:** Party branches and CSR in Chinese private enterprises: moderating effects of political connections.

	(1)	(2)	(3)	(4)	(5)	(6)
	Tool	Tool	Tool	Value	Value	Value
CCP	0.347^***^	0.304^***^	0.346^***^	0.554^***^	0.539^***^	0.498^***^
	(0.054)	(0.061)	(0.062)	(0.051)	(0.057)	(0.058)
CCP×IC		0.135^**^			0.042	
		(0.066)			(0.072)	
CCP×PO			0.003			0.144^**^
			(0.075)			(0.070)
Controls	Yes					
Industry	Yes					
Province	Yes					
Constant	1.832^***^	1.871^***^	1.834^***^	1.261^***^	1.274^***^	1.297^***^
	(0.207)	(0.208)	(0.208)	(0.195)	(0.196)	(0.196)
*N*	5,005	5,005	5,005	5,005	5,005	5,005
*R*-squared	0.133	0.134	0.133	0.267	0.267	0.268

^***^p < 0.01, ^**^p < 0.05.

## 5. Endogeneity and robustness test

### 5.1. Endogeneity test

The article’s most concerning endogeneity is selection bias, where larger firms may perform better in terms of CSR but larger firms are more likely to embed party branches. When the sample exhibits selection bias brought on by quantifiable factors, Propensity Score Matching (PSM) can be utilized to reduce the bias. It selects a firm from the sample that matches other traits with the party branch-containing company’s other traits. The CSR performance of these two companies is then contrasted to determine the real influence of the party branch on CSR.

We used local linear regression, 1:1 matching, radius matching, kernel matching, and propensity score matching to match all of the control variables. We also estimated the treatment effects for propensity score matching. After the balance test, the bias of each variable was also greatly decreased. The largest bias after matching is 3.6%, which is less than 5%. The detailed results of the balance test are not reported in this study due to space constraints.

Table 7 shows that regardless of the matching rule selected, the ATT of the party branch on the CSR performance of private enterprises is significant at the 1% level. The aforementioned findings somewhat reduce the endogeneity brought on by selection bias. It demonstrates the causal link between the party branch and CSR in private enterprises.

**Table 7:** Propensity score matching.

	1:1 matching		radius matching		kernel matching		local linear regression matching		N
	ATT	Value of *t*	ATT	Value of *t*	ATT	Value of *t*	ATT	Value of *t*	
$CSR_{1}$	1.309^***^	5.32	1.034^***^	5.10	1.074^***^	5.41	1.060^***^	4.31	5,005
$CSR_{2}$	2.069^***^	7.01	1.696^***^	7.59	1.756^***^	7.98	1.743^***^	5.90	5,005
$CSR_{3}$	1.137^***^	4.27	1.152^***^	5.54	1.219^***^	5.98	1.197^***^	4.49	5,005

^***^p < 0.01.

Additionally, we reduced endogeneity brought on by self-selection using the Heckman two-step method. As the instrumental variable for party branches, we select the coverage rate of party branches in the industry where the company is located ( CCP−IV ). This is because, whereas a company’s decision to build a party branch is impacted by the industry’s overall situation, a company’s CSR performance is unaffected by the industry’s party branch coverage. By using Probit estimation, we are able to calculate the Inverse Mills Ratio ( $IMR_{i}$ ) in the first step of the Heckman two-step test. The second step of the regression model then includes $IMR_{i}$ as control variables for testing, which reduces endogeneity. The regression coefficients are still positively significant according to the results of the Heckman two-step test, which are shown in Table 8. Additionally, the coefficients significance of CCP−IV are consistent with the results of the above, demonstrating the validity of the study’s findings.

**Table 8:** Instrumental variable test: Heckman two-step model.

	(1)	(2)	(3)
	$CSR_{1}$	$CSR_{2}$	$CSR_{3}$
CCP−IV	0.882^***^	1.313^***^	1.276^***^
	(0.162)	(0.181)	(0.166)
$IMR_{1}$	−14.378^***^		
	(1.451)		
$IMR_{2}$		5.906^***^	
		(1.661)	
$IMR_{3}$			−9.728^***^
			(1.253)
Controls	Yes		
Industry	Yes		
Province	Yes		
*N*	5,005	5,005	5,005

^***^p < 0.01.

### 5.2. Robustness test

We carried out the subsequent robustness tests to confirm the results’ dependability. First, the sample of financial, energy and mining sectors were excluded before analysis. Second, we create a dummy variable $CSR_{i}\_Dum$ for $CSR_{i}$ and assign a value of 1 to samples with above-average CSR performance and a value of 0 to samples with below-average CSR performance. Then use $CSR_{i}\_Dum$ instead of $CSR_{i}$ for the test. The results of all these robustness tests are consistent with the above. The robustness tests’ details are not reported due to space restrictions, but the results are maintained for reference.

## 6. Conclusion and discussion

### 6.1. Conclusion

The embedding of the party branch in private enterprises is a unique feature of the Chinese economic system. This article explores the effect of party branches on the CSR performance of private enterprises in China and makes an effort to explain the mechanisms based on data from the 11th Chinese Private Enterprise Survey (CPES). We found that, first, the embedding of party branches will alleviate information asymmetry, improve the CSR performance of private enterprises in multiple dimensions by enhancing the perception of private enterprises in policy. Second, in various influence pathways, the party branches will enhance the perceptions of policy related to economic interests, which has a more significant impact on enhancing the performance of philanthropic CSR. Further research reveals that business owners’ first-given and later-generated political connections support the party branches’ perception of policies related to economic and social interests, respectively. After endogeneity and robustness are taken into account using propensity score matching and Heckman’s two-step model, the aforementioned conclusions remain valid. This demonstrates that the party branch embedded into private enterprises effectively performs the anticipated “supervisory” and “leadership” roles, which influences the policy perception and enhances CSR performance.

In summary, the findings in this article demonstrate significant role that the Party branch plays in Chinese private enterprises and reveal how the Party branch will encourage private businesses to actively engage in CSR by improving their perceptions of the Party’s policies. With the aid of these findings, we are able to comprehend the effects brought about by the Party branch’s integration with private businesses inside China’s distinctive political and economic systems. And they complement the findings of political economics research on post-communism and emerging economies and provide theoretical references.

### 6.2. Managerial implications

First, for businesses, building political connections is frequently advantageous for corporate growth, especially in emerging economies. Private businesses can increase their perception of policies and improve their performance by forming connections with the government. Even while party branches cannot be established by corporations in the majority of countries, company owners can nonetheless overcome this obstacle by, for instance, developing personal relationships with government officials.

Second, although it appears to be impossible to do in most states, we found that it is feasible for the ruling party to boost the effectiveness of policies by forming party organization in firms. However, the ruling party can still influence businesses by, for example, strengthening their cooperation with them in order to increase their perception of the political party’s policy proposals.

Finally, the public should encourage companies to engage in CSR activities. There is no doubt that CSR has benefited stakeholders, despite some studies showing that it is not driven entirely by altruism. The public can reward companies that demonstrate strong CSR performance to encourage such sustainable business, such as purchasing more of their products and services.

### 6.3. Limitations and future research directions

This study also has some limitations. For businesses, improving CSR performance comes at a high cost. If it goes beyond what is reasonable, it may have a negative impact on the operations and financial condition of the business. This study only reveals the effects and mechanisms of party branch promotion of CSR, but does not provide a comprehensive analysis of the overall impact of this behavior on the company. Despite the social externalities of CSR activities, it is undoubtedly detrimental if firms cater to bureaucracies at the expense of long-term growth under political intervention. For example, party branches may make mandatory donation requests to improve CSR performance, but this does not represent the true will of the companies. Entrepreneurs may also hope to structure political-business relations by conducting good CSR activities, thereby obtaining rent-seeking gains for the firm or seeking higher political status for themselves. In addition, there may also be multiple mechanisms for party branches to promote CSR. However, due to data limitations, this study was not able to analyze other possible mechanisms in detail. We ask these questions in the hope that future research will address them.

## Data availability statement

The raw data supporting the conclusions of this article will be made available by the authors, without undue reservation.

## Author contributions

All authors listed have made a substantial, direct, and intellectual contribution to the work and approved it for publication.

## Funding

This article was funded by the following projects: 1. National Natural Science Foundation of China Youth Project (Project No. 72102088); 2. Outstanding Doctoral Dissertation Publishing Project of National Social Science Fund (Project No. 2021FYB062); 3. Central Socialist University United Front High-end Think Tank Project (Project No.: ZK20210151).

## Conflict of interest

The authors declare that the research was conducted in the absence of any commercial or financial relationships that could be construed as a potential conflict of interest.

## Publisher’s note

All claims expressed in this article are solely those of the authors and do not necessarily represent those of their affiliated organizations, or those of the publisher, the editors and the reviewers. Any product that may be evaluated in this article, or claim that may be made by its manufacturer, is not guaranteed or endorsed by the publisher.
